# Glycosaminoglycans influence enzyme activity of MMP2 and MMP2/TIMP3 complex formation - Insights at cellular and molecular level

**DOI:** 10.1038/s41598-019-41355-2

**Published:** 2019-03-20

**Authors:** Gloria Ruiz-Gómez, Sarah Vogel, Stephanie Möller, M. Teresa Pisabarro, Ute Hempel

**Affiliations:** 1 Structural Bioinformatics, BIOTEC TU Dresden, Tatzberg 47-51, 01307 Dresden, Germany; 2 Medical Department, Institute of Physiological Chemistry, TU Dresden, Fiedlerstraße 42, 01307 Dresden, Germany; 3 Biomaterials Department, INNOVENT e.V., Prüssingstraße 27 B, 07745 Jena, Germany

## Abstract

The extracellular matrix (ECM) is a highly dynamic network constantly remodeled by a fine-tuned protein formation and degradation balance. Matrix metalloproteinases (MMPs) constitute key orchestrators of ECM degradation. Their activity is controlled by tissue inhibitors of metalloproteinases (TIMPs) and glycosaminoglycans (GAG). Here, we investigated the molecular interplay of MMP2 with different GAG (chondroitin sulfate, hyaluronan (HA), sulfated hyaluronan (SH) and heparin (HE)) and the impact of GAG on MMP2/TIMP3 complex formation using *in vitro*-experiments with human bone marrow stromal cells, *in silico* docking and molecular dynamics simulations. SH and HE influenced MMP2 and TIMP3 protein levels and MMP2 activity. Only SH supported the alignment of both proteins in fibrillar-like structures, which, based on our molecular models, would be due to a stabilization of the interactions between MMP2-hemopexin domain and TIMP3-C-terminal tail. Dependent on the temporal sequential order in which the final ternary complex was formed, our models indicated that SH and HA can affect TIMP3-induced MMP2 inhibition through precluding or supporting their interactions, respectively. Our combined experimental and theoretical approach provides valuable new insights on how GAG interfere with MMP2 activity and MMP2/TIMP3 complex formation. The results obtained evidence GAG as promising molecules for fine-balanced intervention of ECM remodeling.

## Introduction

Tissue homeostasis depends on regulated cellular activities strongly affected by the surrounding microenvironment. The composition of extracellular matrix (ECM) has to be adapted to altered physiological situations and conditions. To ensure its integrity, the ECM is constantly remodeled, which requires a fine-tuned balance of protein formation and degradation^1^. Several matrixmetalloproteinases (MMPs) forming a hierarchical activation network and their endogenous inhibitors (tissue inhibitors of matrixmetalloproteinases (TIMPs)) are important players in the highly dynamic ECM remodeling system. Matrix metalloproteinase-2 (MMP2), also named as 72 kDa type IV collagenase and gelatinase A, is distributed in many tissues and associated with several serious diseases. In particular, MMP2 is crucial in cancer cell invasion and for inflammatory bone and joint lesions. Physiologically, MMP2 is mandatory for normal tissue homeostasis e. g. for skeletal, craniofacial development and bone cell growth and proliferation^2,3^. Its proteolytic activity is controlled by the activation of the multi-domain zymogen (proMMP2) form, which is composed of a propeptide (residues 1–80), a catalytic domain (residues 81–192 and 368–436), three fibronectin type 2-like (FNII) domains (residues 199–247, 257–305, 315–363) and a hemopexin (PEX) domain (residues 442–631)^4^. The coordination of Cys73 of the propeptide region to a catalytic zinc ion and the lack of such interaction control the switch from MMP2 inactive to active form, respectively. The catalytic domain, which constitutes the most relevant functional domain, contains an active-site cleft where the substrate binds. FNII domains mediate binding to denatured collagen (gelatin, physiological MMP2 substrate) and are inserted into the catalytic domain. A flexible proline-rich linker connects the C-terminus of the catalytic domain with the PEX domain, which is involved in mediating protein-protein interactions (e.g. to TIMP3 and the membrane type-1 matrix metalloproteinase (MT1-MMP), also known as MMP14) and appropriate substrate recognition, among others^5^. The PEX domain consists of a four-blade propeller structure in which the first and second blades are oriented towards the catalytic domain and to one of the FNII domains.

Proteolytic enzymes like MMP2 are kept in check by endogenous tissue inhibitor of metalloproteinases family (TIMP1–4)^6^. The N-terminal tail of TIMPs binds to the active site of MMPs and, therefore, precludes substrate recognition. TIMP2–4 also participate in the activation of proMMP2 due to a latent activation mechanism that involves the interaction of the TIMP C-terminal tail and the third and fourth blade propellers of the zymogen PEX domain^7^. The resulting complex then localizes at the cell surface where the PEX domain of proMMP2 interacts with the active site of MT1-MMP^5,8,9^.

TIMP1, 2 and 4 have been reported to diffuse in the extracellular environment^6^, whereas TIMP3 is the only member of the TIMP-family that tightly sticks to the ECM^10–12^. This is due to its interaction with sulfated glycosaminoglycans (GAG), e.g. with certain heparan sulfate proteoglycans. GAG are negatively charged polymers that consist of repetitive disaccharide units containing an uronic acid and an amino sugar linked by glycosidic bonds^13^.

GAG have various ECM-related functions including water- and ion-homeostasis, recruitment of several growth factors and ECM proteins and, therefore, they affect signaling pathways and cellular behavior^13^. There are many indications of GAG containing a “code” defined by their chemical structure (e.g. sugar backbone, degree and position of sulfation), which affects various binding partners (extracellular mediators) in a different way^14^. For instance, binding of heparin (HE) to the PEX domain of MMP2 has been reported to promote its autolytic activation^15^. Interestingly, a chemically synthesized high-sulfated hyaluronan derivative (degree of sulfation (D.S.) 3.0) decreased MMP2 activity *in vitro*^16^.

Chemically modified GAG derivatives providing a defined sulfation pattern and chain length constitute attractive molecules for structure-function relationships studies on GAG recognition by MMPs and TIMPs as they minimize batch-to-batch variabilities. For instance, the low-sulfated hyaluronan derivative used here (SH, D.S. 1.2) has a sulfate group at C6-position of the glucosamine as identified by NMR analysis^17^. Previous *in vitro*-studies have demonstrated the exclusive effect of SH on bone remodeling by promoting osteogenic lineage commitment of osteoblast precursor cells^18–23^ as well as by suppressing osteoclast function^21,24^ and inflammatory responses^18,25^. Furthermore, it has been also shown that human bone marrow stromal cells (hBMSC) induce higher amounts of TIMP3 after treatment with SH^26,27^. As a consequence of decreased proteolytic activity, the protein level of several ECM components deposited by hBMSC such as fibronectin was increased^22,27^.

The actual amount of TIMP3 in the ECM is not only a consequence of its formation *per se*, but it is also due to its regulation by the low-density lipoprotein receptor-related protein-1 (LRP-1), which is responsible of binding and endocyting TIMP3^28^. As reported, the endocytosis of TIMP3 via LRP-1 is probably blocked by SH through the competition with LRP-1 for the same TIMP3 recognition region^26^. Atomic-detailed models indicated that sulfated GAG could sequester TIMP3 through binding to multiple sites. However, only one of such binding sites shows a small overlap with MMP2 recognition region, which implies a negligible effect on TIMP3-induced MMP inhibition at high GAG concentration^29^.

The present study investigates the effect of certain sulfated GAG (SH, chondroitin sulfate (CS), and HE in comparison to non-sulfated hyaluronan (HA)) on MMP2 (gene expression, protein level, enzyme activity) and TIMP3 (gene expression, protein level) *in vitro*. MMP2 and TIMP3 were found in the ECM of hBMSC closely and exclusively colocalized with SH, which arises the question whether this interaction may alter proMMP2 activation, MMP2 activity and/or (pro)MMP2/TIMP3-complex formation. Molecular docking and dynamics simulations studies gave insights on how SH and HA may affect MMP2/TIMP3-complex formation. Furthermore, these studies shed light on how the TIMP3-induced MMP2 inhibition is influenced depending on the sequential order in which the interactions in these molecular systems take place. Finally, based on our findings, we propose a molecular mechanism for the colocalization of MMP2 and TIMP3 proteins in the presence of SH.

## Results and Discussion

For the *in vitro* experiments with human bone marrow stromal cells (hBMSC), the synthetic low-sulfated hyaluronan (SH; D.S. 1.2, sulfated at C6 of glucosamine), natural low-sulfated chondroitin sulfate (CS; D.S.0.8) and natural high-sulfated heparin (HE; D.S. 2.2) were used and compared to natural non-sulfated hyaluronan (HA) (see materials and methods) and hBMSC cultures without GAG treatment (Ctrl). Since the commercially available CS is a mixture of chondroitin-4-sulfate (C4S, ca. 70%) and chondroitin-6-sulfate (C6S, ca. 30%), the *in silico* modeling studies were performed with each CS derivative separately.

### Influence of GAG on MMP2 and TIMP3 *in vitro*

**Figure 1:**
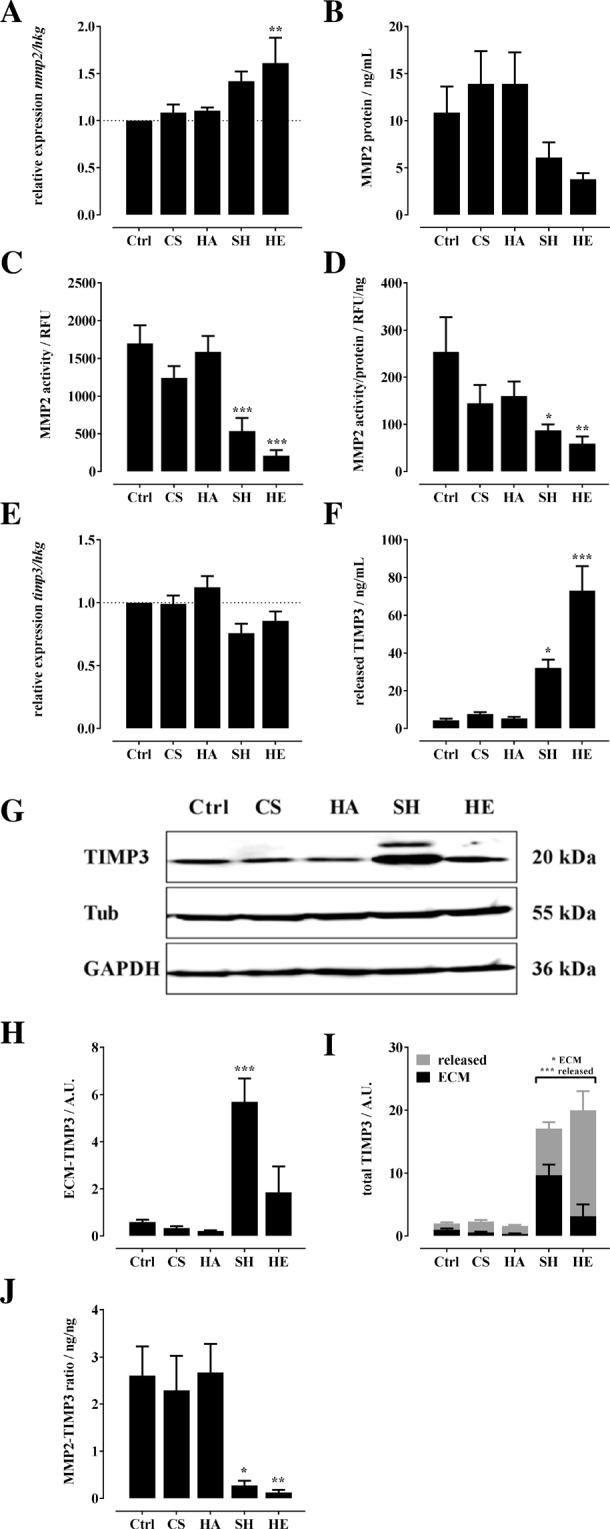
Influence of CS, HA, SH, and HE on MMP2 and TIMP3 formation and MMP2 activity in hBMSC. 7,000 hBMSC/cm^2^ were plated in basic medium and treated with CS, HA, SH, and HE (200 µg/mL each). At day 22 after plating cells and conditioned medium were analyzed. (**A**) *mmp2* expression was assessed by qPCR, normalized to the expression of the house-keeping genes (hkg) *gapdh*, *β-actin*, and *rps26*, and related to untreated control (Ctrl, set to 1) using the comparative quantitation method. Conditioned medium of hBMSC was analyzed for MMP2 protein content using commercially MMP2 ELISA Kit (**B**) and for MMP2 enzyme activity with fluorogenic peptide (MCA-Pro-Leu-Ala-Nva-Dpa-Ala-Arg-NH_2_) as a substrate (**C**). (**D**) The relative MMP2 activity per MMP2 protein amount was calculated from fluorescence signals (MMP2 activity) and ELISA data (MMP2 protein). (**E**) *timp3* expression was assessed by qPCR as described. (**F**) TIMP3 protein amount released into the conditioned medium was determined by ELISA. TIMP3 protein in the pericellular environment of hBMSC was analyzed by Western blotting with chemiluminescence detection. (**G**) shows a representative Western blot (cropped), according full-length blots presented in Supplemental Fig. S3. The chemiluminescence signals of four independent Western blots were quantified densitometrically and normalized to GAPDH and β-tubulin (Tub) as internal loading controls (**H**). (**I**) shows the total TIMP3 content related to Ctrl and the distribution of TIMP3 calculated from Western blot data (pericellular, ECM-associated TIMP3) and ELISA data (released TIMP3). The ratio of released MMP2 protein to released TIMP3 is shown in (**J**). The results are presented as mean ± SEM. Significant differences of treatment vs. Ctrl were analyzed by one-way ANOVA/Bonferroni’s post-test (**A**–**F**,**H**,J), and of SH vs. HE two-way ANOVA/Bonferroni’s post-test (**I**) and are indicated with *(p < 0.05), **(p < 0.01) and ***(p < 0.001), n = 4.

All GAG except HE did not significantly alter the gene expression level of *mmp2* in hBMSC (Fig. 1A). Despite a slight increase in *mmp2* expression by HE, the MMP2 protein content in conditioned medium of hBMSC (released MMP2) was found diminished by SH and HE; the effect, however, was not significant (Fig. 1B). SH and HE significantly reduced MMP2 activity (Fig. 1C). To evaluate whether the decreased MMP2 activity resulted from a decreased amount of enzyme, MMP2 activity was normalized to MMP2 protein. In addition to the reduced MMP2 protein amount, SH and HE significantly reduced MMP2 enzyme activity (Fig. 1D). CS and HA had no significant impact on MMP2 protein and enzyme activity (Fig. 1B–D). Gelatin zymography was used to detect proMMP2 and MMP2 in conditioned medium of hBMSC at day 22 (Supplemental Fig. S1). The densitometric analysis showed that proMMP2 (72 kDa) was not detectable at this time point, but it confirmed the results for MMP2 protein shown in Fig. 1B.

The lower amount of MMP2 in the conditioned medium in the presence of SH and HE is, as seen, not a result of reduced gene expression. This could be caused either by retardation in the ECM, by accelerated degradation of the enzyme, or by forced endocytotic clearance, e. g. via LRP1^30,31^. The observed MMP2 activity could be influenced by proteolytic activation of proMMP2, endogenous or exogenous activators (e. g. zinc ions or mercurial compounds), and natural (e. g. TIMPs) as well as chemical inhibitors. The interaction with diverse ECM proteins as thrombospondin-2 and fibronectin, cell surface receptors and heparan sulfate proteoglycans can further trigger MMP2 protein level, proMMP2 activation and MMP2 activity^30,32^.

Recent proteomics studies^16,27^ reported reduced gelatinolytic activity of MMP2 with osteoblasts cultured on artificial collagen matrices containing high-sulfated hyaluronan (D.S. 3.0), indicating that other sulfated hyaluronan derivatives exhibit comparable effects to SH on MMP2 activity. Van der Smissen *et al*.^33^ showed that MMP1 activity of dermal fibroblasts was decreased by sulfated GAG, indicating that the effects of GAG on MMP are not restricted to MMP2 and to hBMSC.

In the present study we further considered the influence of GAG on TIMP3 *in vitro*. Quantitative PCR analysis exhibit that GAG did not alter the gene expression level of *timp3* in hBMSC (Fig. 1E). TIMP3 protein concentration in the conditioned medium of hBMSC as determined by ELISA was highly induced by SH and HE (Fig. 1F). Compared to Ctrl, CS and HA did not alter TIMP3 concentration (Fig. 1F). The gene expression and protein levels of TIMP1, 2 and 4 (secreted TIMPs) were neither affected by CS, HA, SH nor by HE (Supplemental Fig. S2). TIMP3 content in cell lysates of hBMSC gathering intra- and ECM-associated proteins was analyzed by Western blot (Fig. 1G) and quantified from four independent Western blots by densitometric evaluation of the signals (Fig. 1H; Full length blots in Supplemental Fig. S3). Exclusively SH increased the amount of cell-associated TIMP3 by ca. 10-fold, whereby CS, HA and HE did not significantly affect cell-associated TIMP3 (Fig. 1H). SH and HE increased the total amount of TIMP3 (sum of TIMP3 in conditioned medium and in the cell-associated portion) formed by hBMSC to nearly the same extent but influenced considerably its distribution (Fig. 1I). Whereas in HE-treated hBMSC about 85% of total TIMP3 was released into conditioned medium, in the case of SH-treated cells, about 55% of total TIMP3 was found to stick in the cell-associated portion (Fig. 1I). SH and HE not only decreased MMP2 amount and enzyme activity, increased TIMP3 level and altered its distribution within ECM-associated and released portion, but these two GAG also significantly reduced the MMP2/TIMP3 protein ratio (Fig. 1J). CS and HA had no effect on released and ECM-associated TIMP3 and changed neither TIMP3 distribution nor the MMP2/TIMP3 ratio (Fig. 1F–J).

The significant increased TIMP3 protein level in the presence of SH and HE is not a result of altered gene expression. As discussed for MMP2, SH and HE seem to be responsible for elevated TIMP3 accumulation in the ECM and, probably, TIMP3 is also rescued from endocytotic clearance. Yu *et al*.^10^ showed that several sulfated GAG as HE, heparan sulfate and diverse CS derivatives closely interact with TIMP3. It has been demonstrated that the interaction of sulfated GAG with TIMP3 blocks its binding to the endocytosis receptor LRP-1^12,26,29^. Therefore, it can be assumed that elevated TIMP3 amounts are owed by the stabilizing effect of SH and HE preventing its removal from the system by LRP1 and sequestering it in the ECM. In line with our results, two previous studies described comparable effects of SH on TIMP3^26,27^. A significantly increased TIMP3/MMP2 protein ratio (Fig. 1J) could be one reason for less MMP2 activity in the presence of SH and HE.

**Figure 2:**
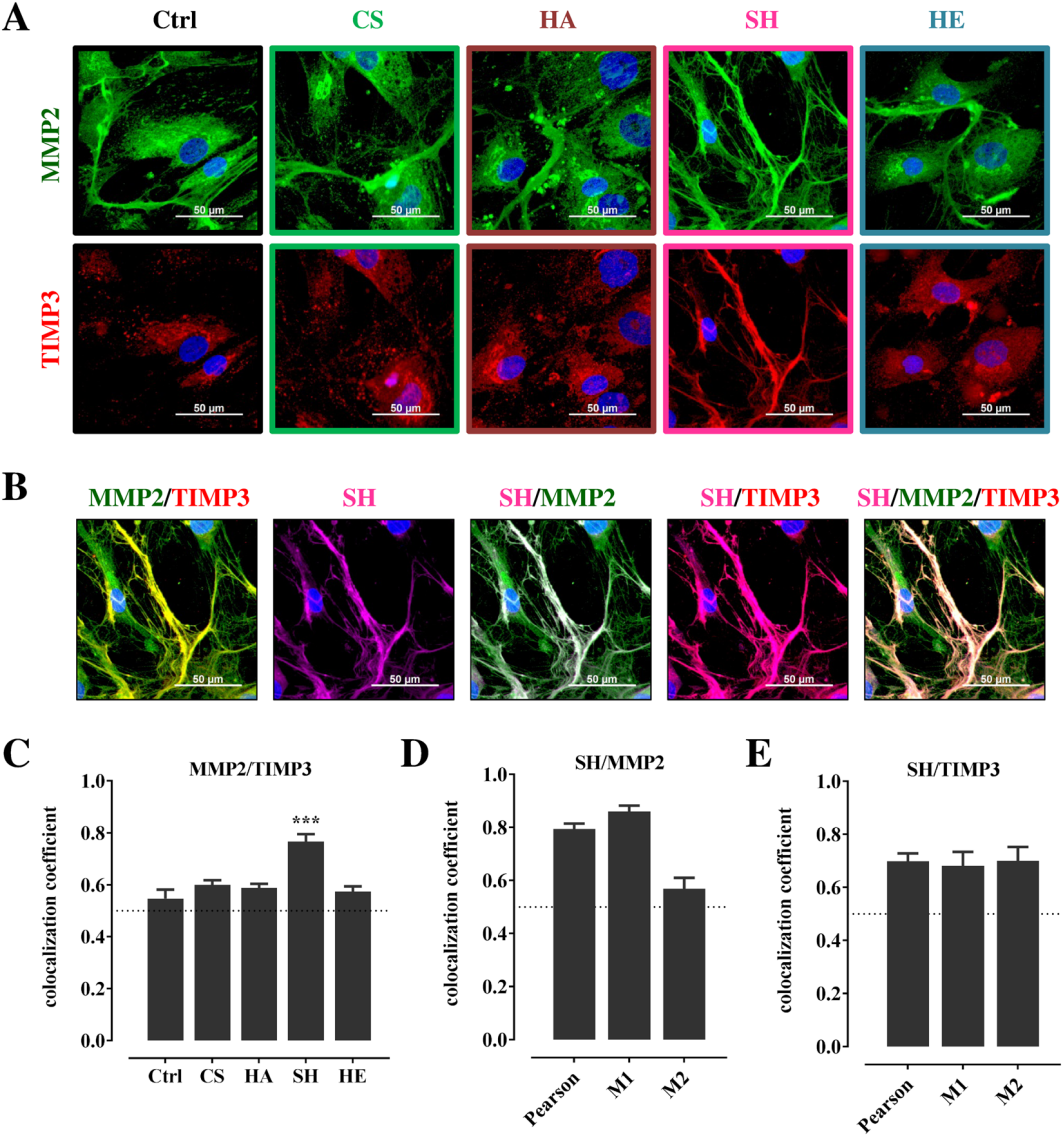
Immunofluorescence staining and colocalization analysis of MMP2 and TIMP3, and of SH with MMP2 and TIMP3. 7,000 hBMSC/cm^2^ were plated in basic medium and treated with CS, HA, SH, and HE (200 µg/mL each) (**A**) or with ATTO655-SH (violet) (**B**). At day 8 after plating cells were fixed with paraformaldehyde and stained for MMP2 (green), TIMP3 (red), and nuclei (blue); scale bars 50 µm (**A**,**B**). (**B**) Images in line from left to right show merged MMP2/TIMP3 (yellow), SH (violet), merged SH/MMP2 (white), merged SH/TIMP3 (purple), and merged SH/MMP2/TIMP3 (pale pink). The extent of colocalization of MMP2 with TIMP3 (**C**) (according to images in **A**) and of SH with either MMP2 (**D**) or TIMP3 (**E**) (according to images in **B**) was calculated as described. Pearson correlation coefficient (summarized signal) values >0.5 (dotted line) indicate a high probability that pixels of both channels are overlay. Manders’ coefficients M1 and M2 (M1 indicates the overlap of SH signal (violet channel) with MMP2 signal (green channel) (**D**,**E**). Values are given as mean ± SEM; significant differences of treatment vs. Ctrl were analyzed by one-way ANOVA/Bonferroni’s post-test and are indicated with ***(p < 0.001), n = 8.

By immunofluorescence staining of CS-, HA-, SH- and HE-treated hBMSC, MMP2 and TIMP3 were visualized (Fig. 2A; Control stainings with only secondary antibodies are depicted in Supplementary Fig. S4). In CS, HA and HE-treated hBMSC, the MMP2 protein, just as TIMP3, was observed in a diffuse pattern around the nuclei, in the perinuclear space and in the cytosol, and it was arranged in a few fibrillar-like structures. Compared to Ctrl and CS-, HA- and HE-treated hBMSC, the morphological arrangement of MMP2 and TIMP3 in the presence of SH was completely different; here both proteins were aligned in fibrillar-like structures. The quantitative analysis of overlapping red and green channel pixels, as verified by Pearson coefficient, indicated only in SH-treated hBMSC a significant colocalization of MMP2 and TIMP3 fluorescence signals aligned in fibrillar-like structures (Fig. 2C).

Although SH and HE increased TIMP3 protein amounts to about the same extent, both GAG substantially differed in their effect on TIMP3 distribution among ECM-bound and released portion (see Fig. 1I). Immunofluorescence images obtained with fluorescent-labelled SH (Fig. 2B) showed that SH itself is part of the MMP2/TIMP3-containing fibrillar-like structures; merged immunofluorescence images showed an overlap of MMP2 and TIMP3, of SH and MMP2, of SH and TIMP3, and hence also of SH, MMP2 and TIMP3. Colocalization analyses gave a Person coefficient of 79% and 70% for the overall overlay of ATTO655-SH with MMP2 and TIMP3, respectively (Fig. 2D). The Manders’ coefficients M1 and M2 indicated that ATTO655-SH colocalized with MMP2 to 86% and with TIMP3 to 68% (M1 value), and that 57% of MMP2 pixel and 70% of TIMP3 pixel overlapped with ATTO655-SH (M2 value) (Fig. 2D–E).

The *in vitro*-experiments with hBMSC demonstrated a substantial impact of SH and HE on the MMP2/TIMP3 system and gave evidence of a very tight vicinity of SH, MMP2 and TIMP3 in fibrillar-like extracellular structures. Whether the highly increased TIMP3 level, or a direct inhibitory/blocking effect of GAG on MMP2 activity, or the modulation of MMP2/TIMP3 complex formation by GAG could be an explanation for the reduced MMP2 activity seen in the presence of SH and HE was taken apart and elucidated in cell-free assays and by molecular modeling.

### Influence of GAG on MMP2 enzyme activity and TIMP3-induced MMP2 inhibition

**Figure 3:**
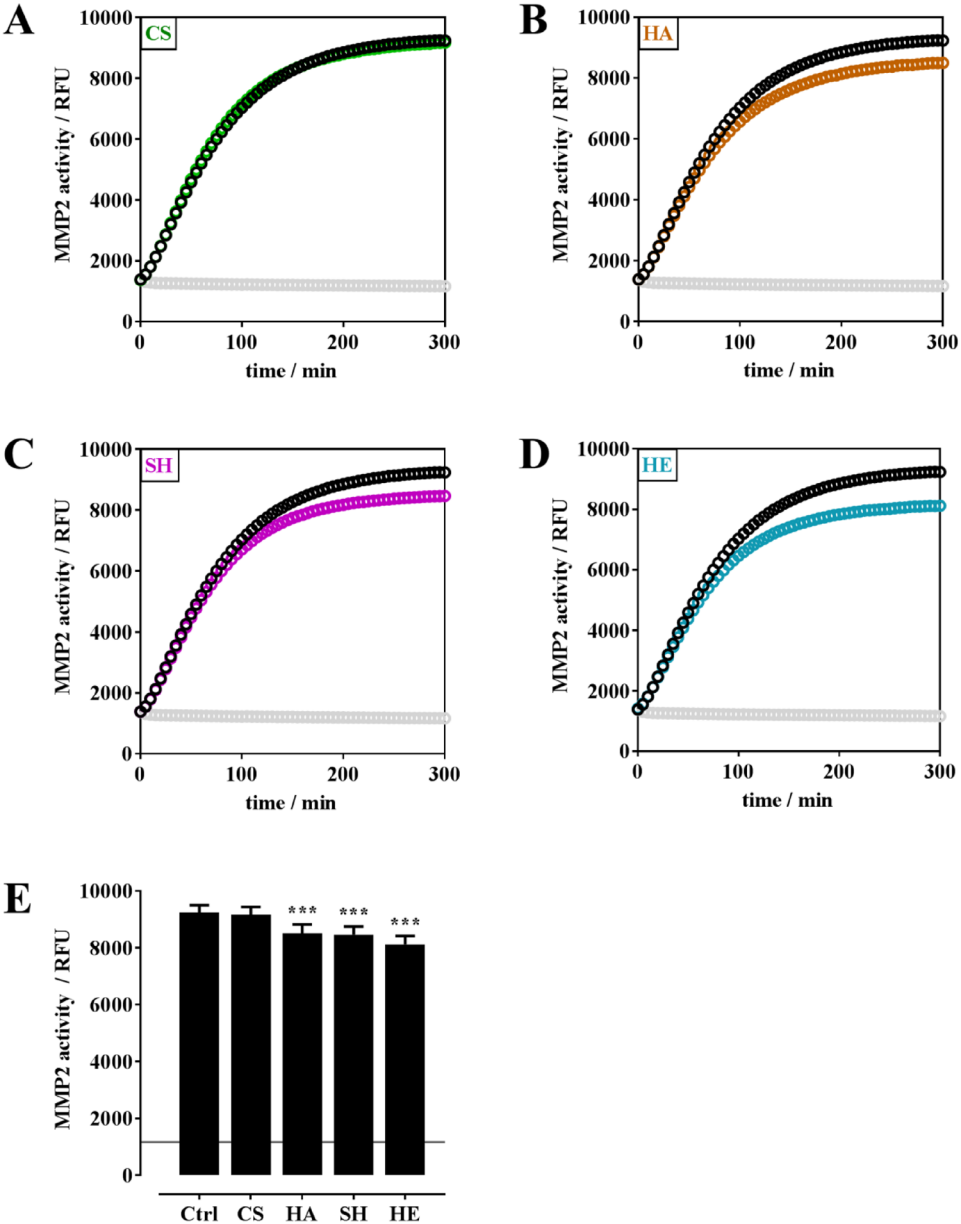
Influence of CS, HA, SH, and HE on MMP2 enzyme activity. MMP2 enzyme activity was determined with rhMMP2 (100 ng/mL) and 50 µM fluorogenic peptide (MCA-Pro-Leu-Ala-Nva-Dpa-Ala-Arg-NH_2_) as a substrate. Kinetic measurement was performed for 5 h without GAG (black curve) and in the presence of CS (**A**, green), HA (**B**, brown), SH (**C**, pink), and HE (**D**, teal) (200 µg/mL each; 20–1,000 µg/mL shown in Supplemental Fig. S5); blanks are given in light grey. (**E**) shows the endpoint values of MMP2 activity after 5 h; the horizontal line indicates the blank value. The results are presented as mean ± SEM. Significant differences of treatment vs. Ctrl were analyzed by one-way ANOVA/Bonferroni’s post-test and are indicated with ***(p < 0.001), n = 4.

The plethora of co-players *in vitro* influencing in parallel MMP2 and TIMP3 makes it difficult to unravel the effects of GAG on MMP2/TIMP3 complex formation. Thus, a cell-free enzyme activity assay with recombinant human MMP2 and TIMP3 and a fluorogenic peptide as a substrate was used to study the influence of CS, HA, SH and HE (200 µg/mL, each) on MMP2 enzyme activity and TIMP3-mediated MMP2 inhibition (Fig. 3A–D). The enzymatic reaction was monitored over 5 h. In the first 120 min the proteolytic activity was independent of GAG. Finally, after 5 h, HA, SH and HE induced a slight but significant ca. 10% decrease in MMP2 enzyme activity (Fig. 3E). Varying the concentration of CS, HA and HE from 20–1,000 mg/mL had no influence on their effects on MMP2 activity (significant differences were only seen for control (0 µg GAG/mL) *vs*. particular GAG concentrations; Supplemental Fig. S5). For SH a marginal dose-dependent effect on MMP2 activity was seen, ranging from 100% for Ctrl (0 µg SH/mL) to 85% of MMP2 activity in the presence of 1,000 µg SH/mL. In a control experiment, the fluorescent peptide released by MMP2 activity from the fluorogenic peptide substrate was incubated with CS, HA, SH and HE to exclude a quenching of the fluorescence signal by GAG (see Supplemental Fig. S6).

**Figure 4:**
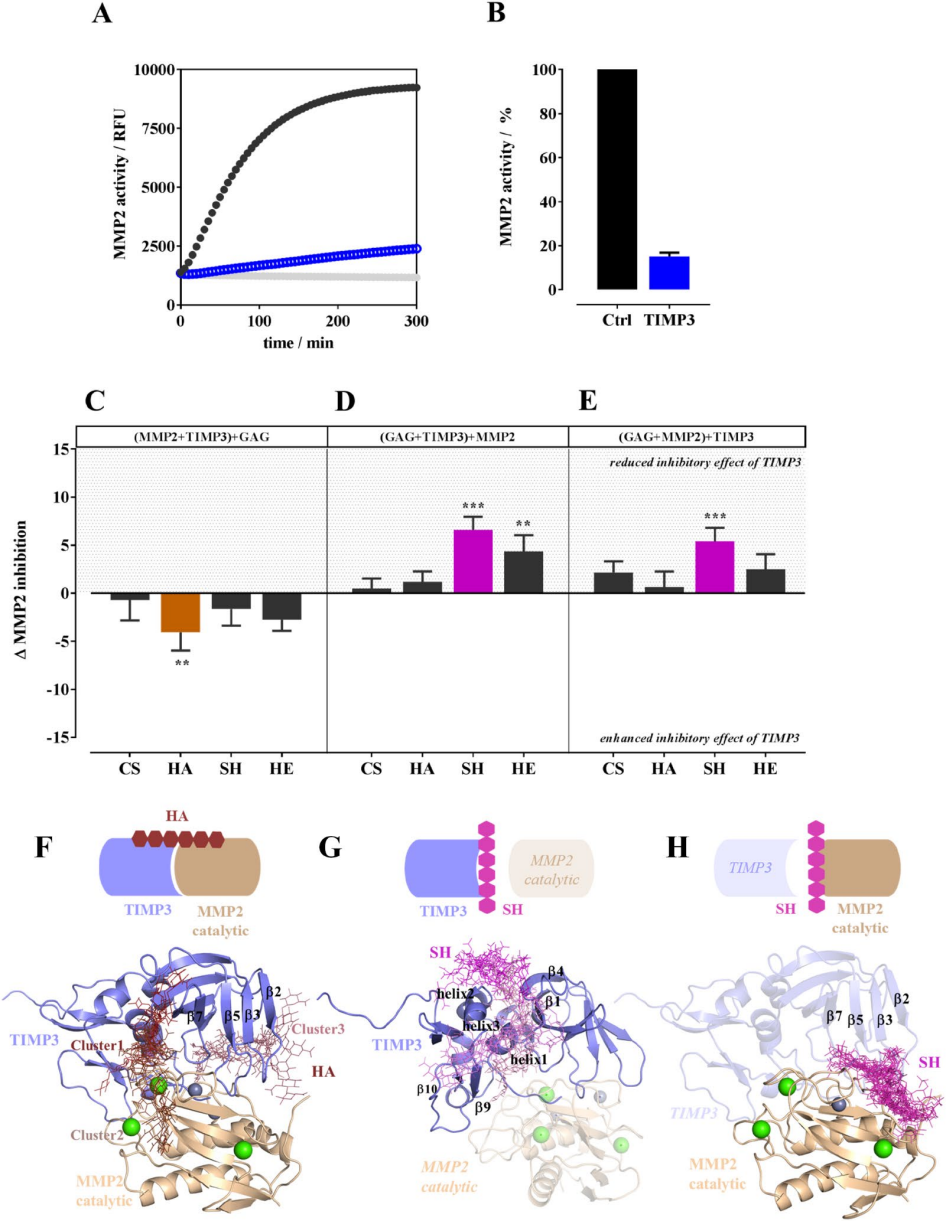
Influence of CS, HA, SH, and HE on TIMP3-induced MMP2 inhibition. MMP2 enzyme activity was determined with rhMMP2 (100 ng/mL) and 50 µM fluorogenic peptide (MCA-Pro-Leu-Ala-Nva-Dpa-Ala-Arg-NH_2_) as a substrate. Kinetic measurement was performed for 5 h without TIMP3 (black curve) and in the presence of 40 ng rhTIMP3/mL (**A**, blue curve); blanks are given in light grey. TIMP3-induced inhibition of MMP2 activity after 5 h (endpoint of kinetic measurement) is given in percent of MMP2 activity (**B**). In the presence of GAG (200 µg/mL) the activity assay was performed in different sequence: either MMP2 and TIMP3 or TIMP3 and GAG or MMP2 and GAG (given in parenthesis) were incubated for 30 min before adding the third component. (**C**–**E**) show how the sequence of incubating the assay components influenced the inhibitory effect of TIMP3 on MMP2 activity. The results are presented as mean ± SEM. Significant differences of treatment vs. control were analyzed by one-way ANOVA/Bonferroni’s post-test and are indicated with *(p < 0.05), **(p < 0.01), and ***(p < 0.001), n = 4. (**F**–**H**) Schematic representation of molecular recognition of GAG to MMP2/TIMP3 complex, TIMP3 and MMP2, respectively, and corresponding docking results using Autodock3 and DBSCAN clustering. MMP2 catalytic domain (PDB ID 1QIB (2.8 Å)) and TIMP3 model are shown in pale and blue cartoon, respectively. TIMP3 model in (**H**) and MMP2 in (**G**) are shown in blue and pale transparency, respectively, just for illustrative purposes, although not taken into account for docking calculations. Calcium and zinc ions are shown in green and grey spheres, respectively. Different clusters of GAG are shown in sticks with color gradient: SH (**G**,**H**, pink) and HA (**F**, brown). The cartoons above illustrate in a schematic manner the recognition of GAG to MMP2/TIMP3 complex, TIMP3 and MMP2 predicted by molecular modeling.

For TIMP3-mediated MMP2 inhibition experiments, MMP2 and TIMP3 were used in a molecular ratio of 1:1 (appx.), which reduces MMP2 activity to about 15% (Fig. 4A,B). The influence of CS, HA, SH and HE on TIMP3-induced inhibition of MMP2 activity was examined in three different settings in which always two of the three components were pre-incubated for 30 min at 25 °C before adding the third one. Figure 4C–E show GAG-induced differences in TIMP3-mediated MMP2 inhibition compared to the control w/o GAG (=15% residual activity of MMP2 in the presence of TIMP3). When the MMP2/TIMP3 complex was formed at first, HA significantly strengthened the TIMP3-induced inhibition of MMP2 activity leading to about 5% less MMP2 activity (Fig. 4C). CS, SH and HE had no influence on the inhibitory efficiency of such a pre-formed MMP2/TIMP3 complex. The pre-incubation of either TIMP3 (Fig. 4D) or MMP2 (Fig. 4E) with CS and HA did not alter the inhibitory effect of TIMP3 on MMP2 activity. In the presence of SH and HE, a weakening of TIMP3 inhibitory efficiency was observed. When either TIMP3 or MMP2 were pre-incubated with SH and when TIMP3 was pre-incubated with HE, the effect was highly significant.

### Molecular recognition of GAG to MMP2/TIMP3, TIMP3, MMP2, proMMP2 and proMMP2/TIMP3

Molecular docking was used to investigate GAG recognition by the MMP2/TIMP3 complex, TIMP3, MMP2, proMMP2 and the proMMP2/TIMP3 complex. The resulting GAG-protein complexes were refined by molecular dynamics (MD) simulations to obtain binding energies and atomic-detailed information about the intermolecular interactions taking place (see materials and methods).

First, the protein structure of MMP2 catalytic domain (PDB ID 1QIB (2.8 Å)) was used to build a three-dimensional atomic model of its complex with TIMP3. For the *in silico* calculations, GAG hexamers HA, SH, HE and CS were used. In particular, for CS, both chondroitin-4- (C4S) and chondroitin-6-sulfate (C6S) were taken into account as the *in vitro* experiments were carried out with a 70:30 mixture of both.

For the pre-formed MMP2/TIMP3 complex, all GAG were predicted to bind to TIMP3. Interestingly, HA was the only GAG disposed along the surface of both proteins (Fig. 4F). After energy refinement by MD simulations, HA was found to slightly strengthen the interaction between both proteins (Table 1, Supplemental Fig. S7) and, thereby, enhancing the ory effect of TIMP3 on MMP2 catalytic domain as stated in the enzyme activity test (Fig. 4C). In the most favorable case (HA-Cluster1), the simultaneous interactions of HA with Arg84 of TIMP3, and Trp147 and Lys158 of MMP2 catalytic domain, among others, could stabilize the MMP2/TIMP3 complex in ca. 4 kcal/mol (ΔG_MMP2/TIMP3_ = −49.5 ± 9.6 kcal/mol, ΔG_MMP2-HA/TIMP3_ = −53.1 ± 9.1 kcal/mol, computed with MM-GBSA^34,35^).

**Table 1:** MM-GBSA binding free energies of MMP2/TIMP3 complex in the absence and presence of HA.

MMP2/TIMP3	ΔG (kcal/mol) mean ± SEM
no HA	−49.5 ± 9.6
HA-Cluster1*	−53.1 ± 9.1
HA-Cluster2*	−46.4 ± 6.3
HA-Cluster3*	−45.1 ± 11.4

*Clusters defined according to Fig. 4F.

Next, recognition of TIMP3 by C4/6S, HA, SH and HE and its possible influence on MMP2 activity was investigated. Binding poses along β1/β4 and helix 2 were obtained for all GAG, which is in concordance with previous findings^29^. For SH, additional binding poses were predicted along helix 1 at the N-terminus and loop regions between helix 3, β9 and β10 at the C-terminal of TIMP3, which partially overlap with the recognition region of MMP2 catalytic domain (Fig. 4G). As observed experimentally, this weakens TIMP3-induced MMP2 inhibition by SH (Fig. 4D).

Molecular docking calculations suggested that all GAG and TIMP3 (loop regions between β2/β3, β5/β6 and β7/β8) could compete for the same recognition region on MMP2 (Fig. 4H, Supplemental Fig. S8). In particular, the GAG-binding region of the MMP2 catalytic domain partially coincides with the substrate-binding cleft site of MMP2^36^. Therefore, also GAG binding might have a negative impact on the MMP2 enzymatic activity, which is in concordance with the experimental observations (Figs 3 and 4).

Besides, due to the fact that the PEX domain of the proMMP2 has been reported to be involved in the activation of the enzyme by interacting with proteins such as TIMP2-4^5,7,9^ and MT1-MMP^5,8,9^, and also with HE^15^, the recognition of GAG by proMMP2 and proMMP2/TIMP3 was further investigated. The proMMP2 structure (PDB ID 1GXD (3.1 Å)) and its modeled complex with TIMP3 (see materials and methods) were used for the *in silico* experiments.

**Figure 5:**
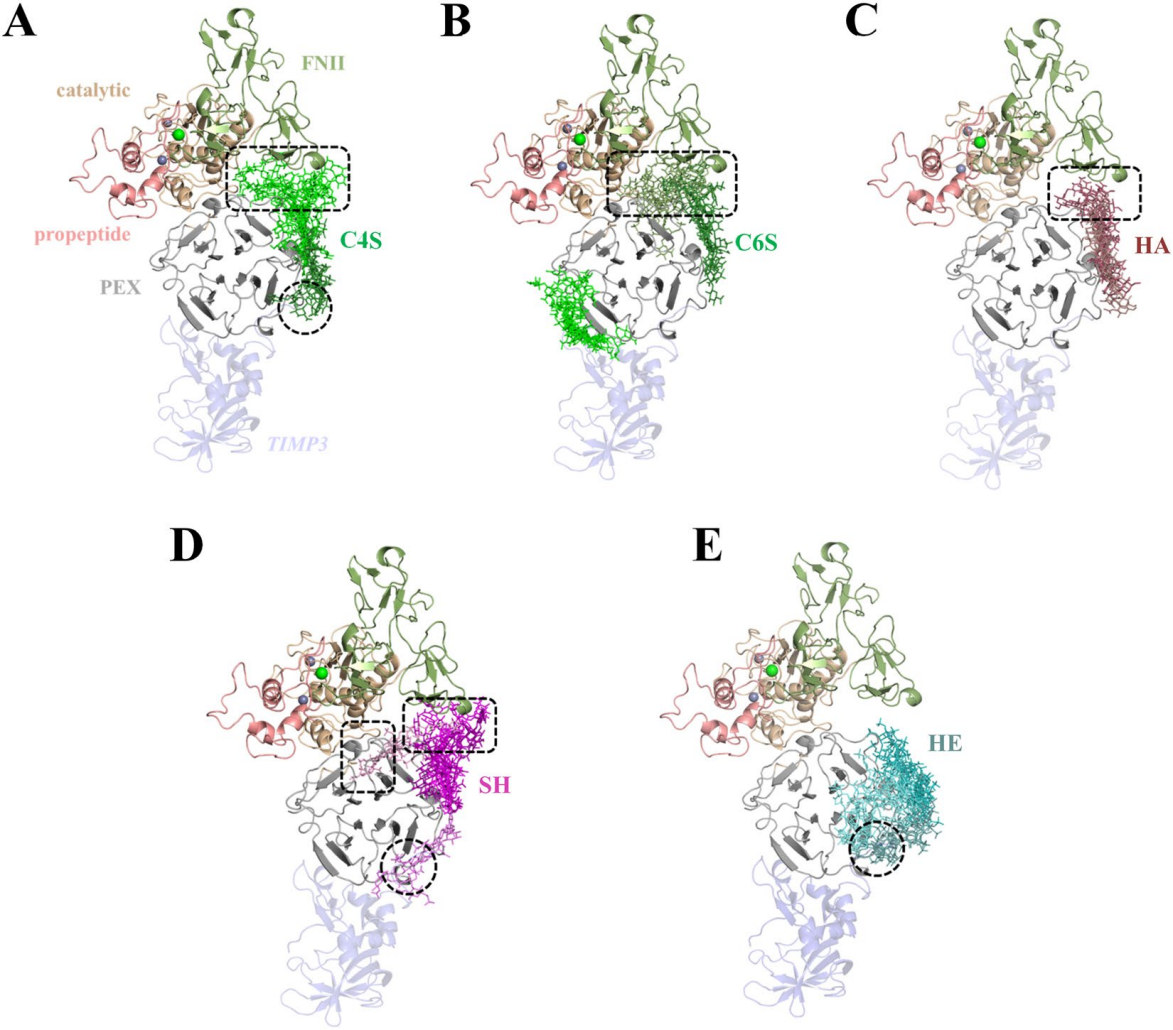
Molecular modeling of GAG in complex with proMMP2. Docking results using Autodock3 and DBSCAN clustering are shown. proMMP2 (PDB ID 1GXD (3.1 Å)) is shown in cartoon and colored by domains: propeptide (light pink), catalytic domain (pale), FNII (green) and PEX domain (grey). Different clusters of GAG are shown in sticks with gradient colors: (**A**) C4S (green), (**B**) C6S (green), (**C**) HA (brown), (**D**) SH (pink) and (**E**) HE (teal). Calcium and zinc ions are shown in green and grey spheres, respectively. The TIMP3 model, although not taken into account for docking calculations, is shown in blue transparency just for illustrative purposes. Discontinuous black circles and rectangles highlight the PEX domain recognition by TIMP3 C-terminal tail and FNII, respectively.

In the recognition studies of proMMP2, C4/6S, HA, SH and HE were predicted to bind to the PEX domain (Fig. 5). Interestingly, SH, HE and, in lesser extent, C4S were found to bind to the same PEX region at the C-terminal tail of TIMP3 (Fig. 5A,D,E), which would preclude PEX/TIMP3 C-terminal tail recognition. In addition, GAG binding poses bridging the PEX and FNII domains were obtained for C4/6S, HA and SH (Fig. 5A–D). Besides, C4/6S and SH adopted poses bridging the PEX and the catalytic domain (Fig. 5A,B,D). The potential interactions of GAG with some regions of FNII and catalytic domain might affect the interaction of proMMP2 with ECM proteins, which are putative substrates for MMP2^5,37^. In conclusion, the interaction of SH and HE with the PEX domain might affect the activation of proMMP2 leading to different MMP2 activity *in vitro* (Fig. 1).

**Figure 6:**
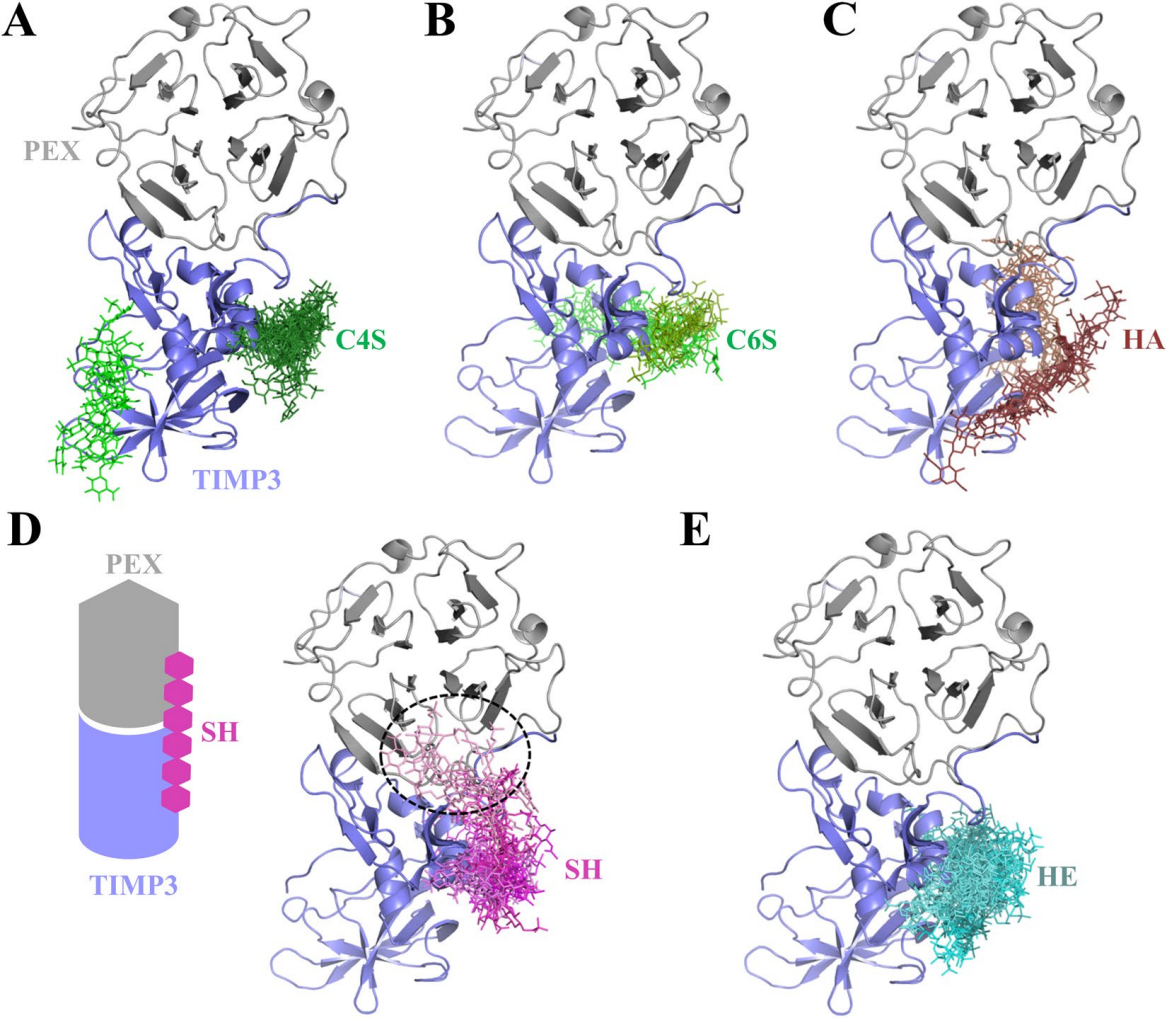
Molecular modeling of GAG in complex with proMMP2(PEX)/TIMP3. Docking results using Autodock3 and DBSCAN clustering of GAG (sticks) to PEX domain of proMMP2 (grey cartoon) (PDB ID 1GXD (3.1 Å)) in complex with TIMP3 model (blue cartoon). Different clusters of GAG are highlighted in gradient colors: (**A**) C4S (green) (**B**) C6S (green), (**C**) HA (brown), (**D**) SH (pink) and (**E**) HE (teal). The cartoon illustrates the molecular bridging of PEX and TIMP3 by SH (discontinuous circle) (**D**).

Molecular docking of GAG to the proMMP2 (PEX domain)/TIMP3 complex (Fig. 6) indicated that C4/6S, HA and HE only recognized TIMP3. Interestingly, only SH was predicted to bridge the C-terminal tail of TIMP3 and the PEX domain of proMMP2 acting as a kind of “molecular glue” (Fig. 6D). Binding energies obtained with the MD-refined ternary complex indicated that the interaction between both proteins in the presence of SH was further stabilized in ca. 10 kcal/mol (ΔG_PEX/TIMP3_ = −73.1 ± 8.8 kcal/mol; ΔG_PEX/TIMP3-SH_ = −82.1 ± 2.2 kcal/mol). PEX domain residues Lys547, Asn548, Arg561, Asn582, Ile584, Gln614, Ser615, Leu616 and Lys617 increased binding strength to TIMP3 in the presence of SH (Supplemental Fig. S9). These data strongly support the results from immunofluorescence staining and colocalization analysis in which TIMP3 and MMP2 were found colocalized exclusively in the presence of SH (Fig. 2A), and where SH was seen to be part of the MMP2/TIMP3 fibrillar-like structures (Fig. 2B).

In this study, the effect of HA, SH, HE and CS on ECM remodeling by the means of gene expression, protein levels and function of MMP2 and TIMP3 in hBMSC was investigated. The expression of *mmp2* and *timp3* was not significantly influenced by CS, HA, SH, and HE. Both, SH and HE significantly decreased MMP2 protein content and additionally reduced MMP2 enzyme activity. This effect could be caused by direct interaction of MMP2 and/or proMMP2 with SH and HE, which in principle might shield the catalytic site or block the activation of the proenzyme. The latter could be neither excluded nor confirmed *in vitro* since proMMP2 was under the detection limit of gelatin zymography. Another reason, which is supported by experimental data, could be the elevated TIMP3 protein levels induced by SH and HE. Nevertheless, both GAG have the same effect on MMP2 and TIMP3 protein activity, but they distinguished themselves in their respective effects on TIMP3 distribution within the ECM. The tight vicinity of MMP2 and TIMP3 obtained only in the presence of SH, as seen in immunofluorescence staining, was the trigger to study at molecular level the interaction of single components in the ternary system of GAG/MMP2/TIMP3 using a combination of a simplified cell-free assay involving recombinant human proteins and molecular docking calculations. Recent proteomics studies have shown that SH and HE, in comparison to other GAG, alter MMP2 and TIMP3 not only in hBMSC and in their pericellular space, but also in hBMSC-derived matrix vesicles^38^. Opposite effects of HE and HA and C6S as well on MMP2 activity are described for human dermal fibroblasts^39^ and for corneal explant cultures^40,41^, respectively. One consequence of the diminished MMP2/TIMP3 ratio and the reduced MMP2 activity is a substantial stabilization and accumulation of several ECM proteins (e.g. fibronectin), in particular in the presence of SH^22,27^.

In a previous study, Rother *et al*.^29^ reported that the interaction of native and chemically modified GAG with TIMP3 had a negligible influence on MMP1 and MMP2 recognition and enzyme activity. In our studies, we observed that the temporal sequence in which the ternary complex of GAG/MMP2/TIMP3 is built determines the impact of GAG on TIMP3-induced MMP2 activity, especially for HA, SH and HE. These results are in good agreement with previous findings on the interaction of TIMP3 with MMP2 and MMP9^42^. To unravel the molecular mechanisms underlying GAG-mediated TIMP3 function, *in silico* docking and MD simulations were performed. The inhibitory capacity of TIMP3 was weakened when either the complex of SH with MMP2 or with TIMP3 was pre-formed, while HA was able to slightly enhance MMP2 inhibition by TIMP3 in the preformed complex between both proteins. Hence, SH is able to impair TIMP3/MMP2 recognition, whereas HA could slightly stabilize the interaction between both proteins once the complex is formed.

In addition, the interplay of GAG on the molecular recognition of proMMP2 and its complex with TIMP3 was examined. Morgunova *et al*.^7^ reported that the MMP2 PEX domain is necessary for the first activation cleavage of proMMP2 and recognition by TIMP2, TIMP3 and TIMP4 in an alternative activation mechanism of proMMP2 in cells. In particular, the interaction of the C-terminal tail of TIMP2 with the MMP2 PEX domain has been proven to be crucial to initiate this latent activation mechanism^9,43^. Therefore, we investigated *in silico* whether different GAG could preclude TIMP3 recognition and modulate cell-mediated proMMP2 activation, as well as their interplay once the proMMP2/TIMP3 complex is formed. Our findings suggested that SH, HE and in certain extent C4S could preclude TIMP3 C-terminal tail binding. It has been previously reported that HE and C4S bind to the PEX domain and promote proMMP2 activation by autolysis^15^ and by cell-bound membrane type-3 MMP (MT3-MMP)^44^, respectively. Taking into account the role of TIMP3 in assisting the localization of proMMP2 on the cell surface previous activation of the zymogen, GAG could play a similar role on the activation of proMMP2. Our molecular models indicated that SH could act as a “molecular glue” by strengthening the interaction between the PEX domain of proMMP2 and the C-terminal tail of TIMP3, which could efficiently assist the colocalization of both proteins as experimentally demonstrated in fluorescence images.

## Conclusions

The results obtained from our combined experimental and molecular modeling approach provide very valuable new insights on how GAG can influence ECM remodeling via interactions with TIMP3, MMP2 or both simultaneously. SH and HA could tightly modulate TIMP3-induced MMP2 inhibition through precluding or supporting the interactions between both proteins, respectively, in a temporal sequential manner. In addition, it was shown that only SH and none of the other GAG (HA, HE or CS) supported the alignment of TIMP3 and MMP2 in hBMSC. Recent studies gave evidence that SH is the only GAG that significantly enhances osteogenic differentiation of hBMSC^19,45^. It still remains to be elucidated whether any link between the two facts (enhanced osteogenic differentiation by SH, increased ECM stability by SH) exists. Taken altogether, defined GAG are able to fine-tune the MMP2/TIMP3 system and so allow a discreet adjustment of ECM remodeling. Through the establishment of atomic-detailed three-dimensional molecular models, and in combination with experimental work, we have been able to provide cellular and mechanistic insights on GAG recognition by TIMP3 and the proMMP2 PEX domain, as well as to characterize their functional impact on MMP2 activity.

## Methods

### Preparation of GAG derivatives

Chondroitin sulfate (CS, mixture of C4S and C6S^46^, from bovine trachea) was obtained from Kraeber (Ellerbek, Germany), heparin (HE, from porcine intestinal mucosa) from Sigma (Taufkirchen, Germany), hyaluronan (from *Streptococcus*, MW 1.100.000 g/mol) from Aqua Biochem (Dessau, Germany).

**Figure 7:**
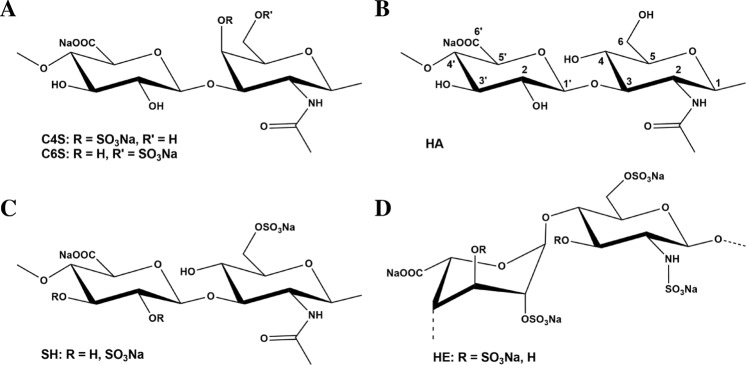
Chemical structure of GAG derivatives. Disaccharide units (Na-salts) of (**A**) chondroitin-4-sulfate (C4S) and chondroitin-6-sulfate (C6S) (D.S. 0.8), (**B**) hyaluronan (HA), (**C**) low-sulfated hyaluronan (SH, D.S. 1.2) and (**D**) heparin (HE, D.S. 2.2).

Low molecular weight hyaluronan (HA) was produced by thermal degradation^47^. Low-sulfated hyaluronan (SH) was synthesized as described^19,22^. Fluorescence labelling of SH was carried out by side-on functionalization with ATTO655-NH_2_ resulting in a dye content of 0.41 µg/mg^22,48^. Chemical structures of GAG are shown in Fig. 7. GAG derivatives have the following characteristics (D.S. (degree of sulfation, number of sulfate residues per disaccharide unit), PD (polydispersity index) calculated from refraction index (RI), MW (molecular weight determined by Laser Light Scattering (LLS)): CS (D.S. 0.8, PD 1.6, MW 20,000 g/mol), HA (no sulfation, PD 2.2, MW 20,000 g/mol), SH (D.S. 1.2, PD 1.8, MW 20,000 g/mol), HE (D.S. 2.2, PD 1.5, MW 17,500 g/mol).

### Cultivation of hBMSC

hBMSC were isolated from bone marrow aspirates^49^ collected from healthy Caucasian donors (males, average age 32 ± 0.4 yrs.) at the Dresden Bone Marrow Transplantation Centre of the University Hospital Carl Gustav Carus. The study was approved by the local ethics commission of Technische Universität Dresden (ethics vote no. EK263122004, EK114042009) and the donors were informed about the procedures and gave their full consent for participation in this study. Methods were carried out in accordance with the relevant guidelines (good scientific according to guidelines of German Research Foundation). hBMSC preparations of individual donors were not pooled and used in passage 3–6.

For the experiments, 7,000 hBMSC/cm^2^ were plated in basic medium (Dulbecco’s modified essential medium (DMEM, 1 g glucose/l) with 10% heat-inactivated fetal calf serum and 20 U penicillin/20 µg streptomycin/mL) on tissue culture polystyrene (TCPS). 24 h after plating, hBMSC were treated with GAG derivatives, 200 µg/mL each.

Medium change and adding of GAG was carried out twice a week. Cells were prepared according to the assay procedures; the conditioned medium was stored at −20 °C until analyses.

### Quantitative PCR analyses

For the analysis of gene expression, RNA was directly prepared from cell lysates and real time PCR analysis was performed as previously described^26^. Primers were synthesized by Eurofins MWG Operon for β-actin, gapdh, rps26^26^, mmp2 (88 bp, 5′-3′-sequences: forward agaaggctgtgttctttgcag, reversed aggctggtcagtggcttg) and timp3 (101 bp, 5′-3′-sequences: forward gtgcaacttcgtggagaggt, reversed agcaggacttgatcttgcagt). The relative expression values were calculated using the comparative quantification method of the RotorGene software release 6.0. For quantitation, values are normalized to the house-keeping genes and related to Ctrl.

### ELISA

MMP2 and TIMP3 protein concentration in conditioned medium of hBMSC was determined using DuoSet ELISA development kits (DY902 for MMP2, and DY973 for TIMP3, BioTechne, Wiesbaden, Germany) according to manufacturer’s instructions. If required, samples were diluted in 1% of bovine serum albumin (BSA, from Calbiochem/Merck, Darmstadt, Germany) in phosphate buffered saline (PBS).

### MMP2 enzymatic activity

Fluorometric MMP2 enzymatic activity assay was performed accordingly to^50^. In general, 20 µL of sample (conditioned medium or rh-proMMP2 (BioTechne, 200 µg/mL in 20 mM Tris-HCl, 150 mM NaCl, 5 mM CaCl_2_, and 0.05% Brij-35^TM^ were diluted in assay buffer (50 mM Tris-HCl, 10 mM CaCl_2_, 175 mM NaCl, 1% dimethyl sulfoxide (DMSO), and 0.05% Brij-35^TM^, pH 7.5) to 500 ng/mL, in the assay a final concentration of 100 rh-proMMP2 ng/mL was applied) were mixed with 20 µL of 1.25 mM 4-aminophenylmercuric acetate (APMA, proMMP2 activator, Sigma, diluted in assay buffer from 1 M stock solution in DMSO to 1.25 mM, final concentration in the assay 0.25 mM) in a black 96 well plate for 20 min at 25 °C. Further, depending on the test set-up, either 20 µL of GAG (diluted with assay buffer, final concentration in the assay 200 µg/mL) or 20 µL of rhTIMP3 (BioTechne, 100 µg/mL in water, diluted with assay buffer, final concentration 40 ng/mL), and 20 µl of assay buffer, or 20 µl of both rhTIMP3 and GAG, or 40 µl of assay buffer were added. In particular tests, the order of adding of the components was varied: For that either rhMMP2 and rhTIMP3, or rhMMP2 and GAG, or rhTIMP3 and GAG were pre-incubated for 30 min at 25 °C before adding the other components. To study the dose-dependent influence of GAG on MMP2 activity, the concentration of GAG was varied from 20–1000 µg/mL, each in 20 µl (see Supplemental Fig. S5). In each case the enzyme reaction was started by adding 20 µL of fluorogenic MMP2 peptide substrate (MCA-Pro-Leu-Ala-Nva-Dpa-Ala-Arg-NH_2_, Calbiochem/Merck, diluted in assay buffer from 1 mM stock solution in DMSO to 50 µM, final concentration 10 µM). The assay blank was made from 80 µL of assay buffer and 20 µL of substrate solution. The fluorescence signal detection was performed in a fluorimeter plate reader (Fluorostar Galaxy, BMG LABTECH, Ortenberg, Gemany) at 320 nm excitation and 405 nm emission in kinetics mode for 300 min in total with 5 min measurement intervals at 37 °C. To exclude that GAG quenched the fluorescence signal, 20 µl of OMMNIMMP fluorogenic control peptide (MCA-Pro-Leu-OH, Enzo Life Science Lörrach, Germany, diluted in assay buffer from 1 mM stock solution in DMSO to 50 µM, final concentration 10 µM) were mixed with 20 µl of GAG, and 60 µl of assay buffer and measured as described above.

### Western blot analysis

Western blot analysis of cell-associated proteins (protein lysis buffer: 6 M urea, 2 M thiourea, 100 mM NH_4_HCO_3_, pH 8) was performed as previously described^22^. The SDS-PAGE (4% stacking gel, 5–15% resolving gel) was run and the proteins were transferred to nitrocellulose membranes (GE Healthcare, Freiburg Germany) by semi-dry blotting. Several proteins were analysed on one blot by separate incubation with mouse anti-hTIMP3-IgG (BioTechne), anti-hGAPDH-IgG (Calbiochem/Merck) or mouse anti-β-tubulin-IgG (Sigma) followed by immunoreaction with horseradish peroxidase (HRP)-conjugated horse anti-mouse-IgG (CST, via New England Biolabs, Frankfurt, Germany). Visualization of immune complex was performed by enhanced chemiluminescence detection (GE Healthcare) using a CCD camera system (MF-ChemiBIS1.6 via Biostep Jahnsdorf, Germany). For densitometric evaluation ImageQuant TL software (GE Healthcare) was used.

### Immunofluorescence staining

Immunofluorescence staining was performed as described before^22^. Fixed hBMSC were incubated with goat anti-hMMP2-IgG (BioTechne) or mouse anti-hTIMP3-IgG (BioTechne) followed by incubation with AlexaFluor488 rabbit anti-goat-IgG (Invitrogen, Karlsruhe, Germany) or AlexaFluor568 goat anti-mouse-IgG (Invitrogen). Nuclei were stained with 0.2 µg 4′,6-diamidino-2-phenylindole (DAPI)/mL and finally samples were embedded in Mowiol 4–88 (Sigma). An Olympus IX70 microscope (Carl Zeiss, Oberkochen, Germany) was used for visualization. Digital images were obtained with an AxioCam MRm camera (Carl Zeiss) using AxioVision software release 4.9 (Carl Zeiss).

For visualization of colocalization of SH/MMP2/TIMP3, hBMSC were incubated with ATTO655-labeled SH (200 µg/mL).

Colocalization analysis of immunofluorescence signals was performed as previously described^22^. The immunofluorescence signals of ATTO655-SH and MMP2 respectively TIMP3 or MMP2 and TIMP3 were quantified with software Fiji setting threshold automatically by Otsu filter. Colocalization values (Pearson, Manders’ coefficients) were calculated from eight representative images using the colocalization plugin JACoP (Just another colocalization plugin)^51^.

### Statistics

All *in vitro*-experiments were performed with four biological independent samples (donors). Fluorometric MMP2 activity determination was performed in quadruplets in four independent experiments. All statistical analyses were performed with GraphPad Prism 7.02 software. Statistical significance was analyzed by one- or two-way ANOVA with Bonferroni’s post-test. More detailed information about the applied tests is given in the figure captures.

### Computer-based modeling and simulation

#### Comparative modeling

Comparative modeling was used to model TIMP3 in complex with proMMP2 and MMP2. TIMP3 N-terminal (res. 1–121) was taken from PDB (PDB ID 3CKI (2.3 Å)). TIMP3 C-terminal (res. 122–186) was modeled using as template the structure of TIMP-2/proMMP2 complex (42% identity and 69% similarity between TIMP2 and 3) (PDB ID 1GXD (3.1 Å)) with Modeller in Discovery Studio (Accelrys)^52^. The TIMP3-MMP2 complex was built using the TIMP3 model and the structure of MMP2 (PDB ID 1QIB (2.8 Å)) docked using as template the structure of MMP13 in complex with TIMP2 (PDB ID 2E2D (2.0 Å)). MMP2 and MMP13 catalytic domains share 65% identity and 76% similarity. proMMP2, MMP2, TIMP3, proMMP2/TIMP3 and MMP2/TIMP3 were minimized in explicit water previous to docking studies (see molecular dynamics simulations section for details).

#### Molecular docking

Docking was carried out with Autodock 3^53^ to predict binding of GAG hexamers (HA, SH (sulfated at C6 of each N-acetylglucosamine saccharide unit), HE and CS (either sulfated at C4 or C6 of N-acetylgalactosamine, specified here as C4S and C6S, respectively, see formulas in Fig. 7). Autogrid3 was used to calculate the atomic potential of each structure covering the full surface (proMMP2: 120 Å × 126 Å × 120 Å grid box and 0.775 Å spacing grid, MMP2: 126 Å × 126 Å × 126 Å and 0.390 Å, TIMP3: 126 Å × 126 Å × 126 Å and 0.450 Å, proMMP2/TIMP3: 80 Å × 110 Å × 126 Å and 0.800 Å, MMP2/TIMP3: 126 Å × 124 Å × 126 Å and 0.500 Å. GAG were treated flexible and proteins rigid. 1,000 independent runs were carried out with the Lamarckian genetic algorithm (initial population size: 300, termination condition: 10,000 generations, energy evaluations: 9,995 × 10^5^). The top 50 docking solutions were clustered with DBSCAN as previously described^54^. Four representative poses from each cluster were taken for further refinement.

#### Molecular dynamics simulations

The complexes proMMP2(PEX domain)/TIMP3, MMP2/TIMP3 and the selected from the docking studies were further refined by Molecular Dynamics (MD) simulations in AMBER14^55^. GAG charges and parameters were taken from GLYCAM 06-j^56^. Charges for sulfates were taken from literature^57^. Protein parameters were obtained from ff14SB^55^. Each GAG/protein complex was solvated in a TIP3P truncated octahedral box and neutralized with Cl^−^ counterions. MD simulations were preceded by two energy-minimization steps: i) only solvent and ions were relaxed with position restraints for solute (500 kcal/mol·Å^2^) by 1000 steps of steepest descendent followed by 500 of conjugate gradient; ii) the entire system without restraints with 3000 cycles of steepest descendent and 3000 of conjugate gradient. The system was heated up from 200 K to 300 K in 20 ps with 10 kcal/mol·Å^2^ position restraints. Langevin temperature coupling with a collision frequency γ = 1 ps^−1^ was used. Three equilibration steps of 500 ps each with 10, 5, and 2 kcal/mol·Å^2^ consecutively decreased position restraints for the Zn^2+^ and Ca^2+^ were applied when considering MMP2. Isothermal-isobaric ensemble (NPT) with Langevin thermostate and Berendsen barostat were applied under periodic boundary conditions. The system was further equilibrated 500 ps without restraints at 300 K under same conditions. 20 ns simulation was carried out at 300 K NPT conditions. SHAKE was used to constrain all bonds involving hydrogens with a time step of 2 fs per step. 8 Å cutoff was used for nonbonded interactions, and PME was used to treat long-range electrostatic interactions. MD trajectories were recorded every 10 ps. GAG pyranose rings were harmonically restrained. Trajectories were visualized in VMD^58^ and intermolecular H-bonds evaluated with CPPTRAJ in AMBER. At least 10% occupancy was taken as H-bond formation criterion. Energy decomposition per residue, pairwise and binding free energy analysis of 200 simulation frames were obtained with MM-GBSA (gb = 2) in AMBER14^34,35^. R^59^ was used for data analysis and PyMOL^60^ for preparation of figures.

## Supplementary information

Supplementary information

## Data Availability

The datasets generated and analyzed during the current study are available from the corresponding author on request.
