# Supplementary material for: Glycosaminoglycans influence enzyme activity of MMP2 and MMP2/TIMP3 complex formation - Insights at cellular and molecular level
Source: Sci Rep. 2019 Mar 20;9:4905. doi: 10.1038/s41598-019-41355-2 (PMC6426840; doi:10.1038/s41598-019-41355-2)
Supplement: Supplementary file 1 — Supplementary information [file 41598_2019_41355_MOESM1_ESM.docx]

**Glycosaminoglycans influence enzyme activity of MMP2 and MMP2/TIMP3 complex formation - Insights at cellular and molecular level**

Gloria Ruiz-Gómez^1#^, Sarah Vogel^2#^, Stephanie Möller^3^,

M. Teresa Pisabarro^1§^, Ute Hempel^2§*^

^1^Structural Bioinformatics, BIOTEC TU Dresden, Tatzberg 47-51, 01307, Dresden, Germany.

^2^ Medical Department, Institute of Physiological Chemistry, TU Dresden, Fiedlerstraße 42, 01307, Dresden, Germany.

^3^Biomaterials Department, INNOVENT e.V., Prüssingstraße 27 B, 07745, Jena, Germany.

^#/§^These authors contribute equally to this work.

*Corresponding author: ute.hempel@tu-dresden.de

**Supplementary material**

**Materials and methods**

**Zymography**

For analysis of proMMP2 and MMP2 in conditioned media, samples were separated in a 7.5%ic SDS-polyacrylamide gel containing 0.05% gelatine as described ^1,2^. After three-times washing in 2.5%ic aqueous Triton X-100 and over-night incubation in 50 mM Tris-buffer, pH 7.4 supplemented with 0.1% Triton X-100, 5 mM CaCl_2_ and 1 µM ZnCl_2_ at 25°C the gel was stained with Coomassie blue R250. Gelatine-free, unstained bands indicating gelatinolytic activity (72 kDa=proMMP2, 66 kDa=MMP2) were quantified using ImageQuant TL software (GE Healthcare).

**Supplemental Figures**

**
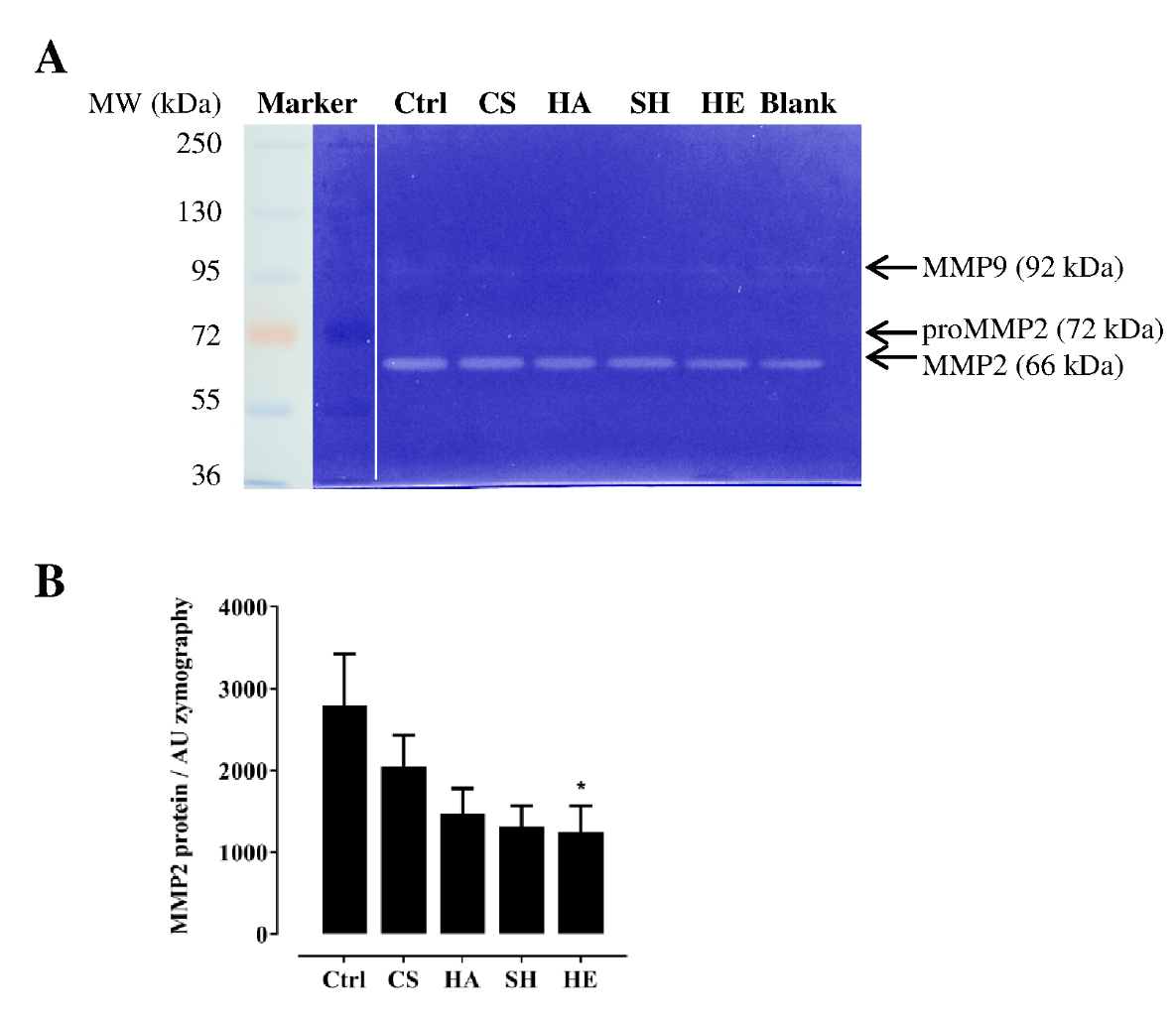
**

**Fig. S1: Influence of CS, HA, SH, and HE on MMP2 activation in hBMSC.**

7,000 hBMSC/cm² were plated in basic medium and treated with CS, HA, SH, and HE (200 µg/mL each). At day 22 after plating conditioned medium was analyzed for MMP2 activity using gelatine zymography. Basic medium was used as Blank. A representative zymogram (full length) visualizing proMMP2 (72 kDa) and MMP2 (66 kDa) is shown in (**A**). The amount of MMP2 in conditioned medium (white bands) was calculated densitometrically (**B**). Results are presented as mean ± SEM. Significant differences of treatment vs. Ctrl were analyzed by one-way ANOVA/Bonferroni’s post-test and are indicated with * (p<0.05), n=4.

**
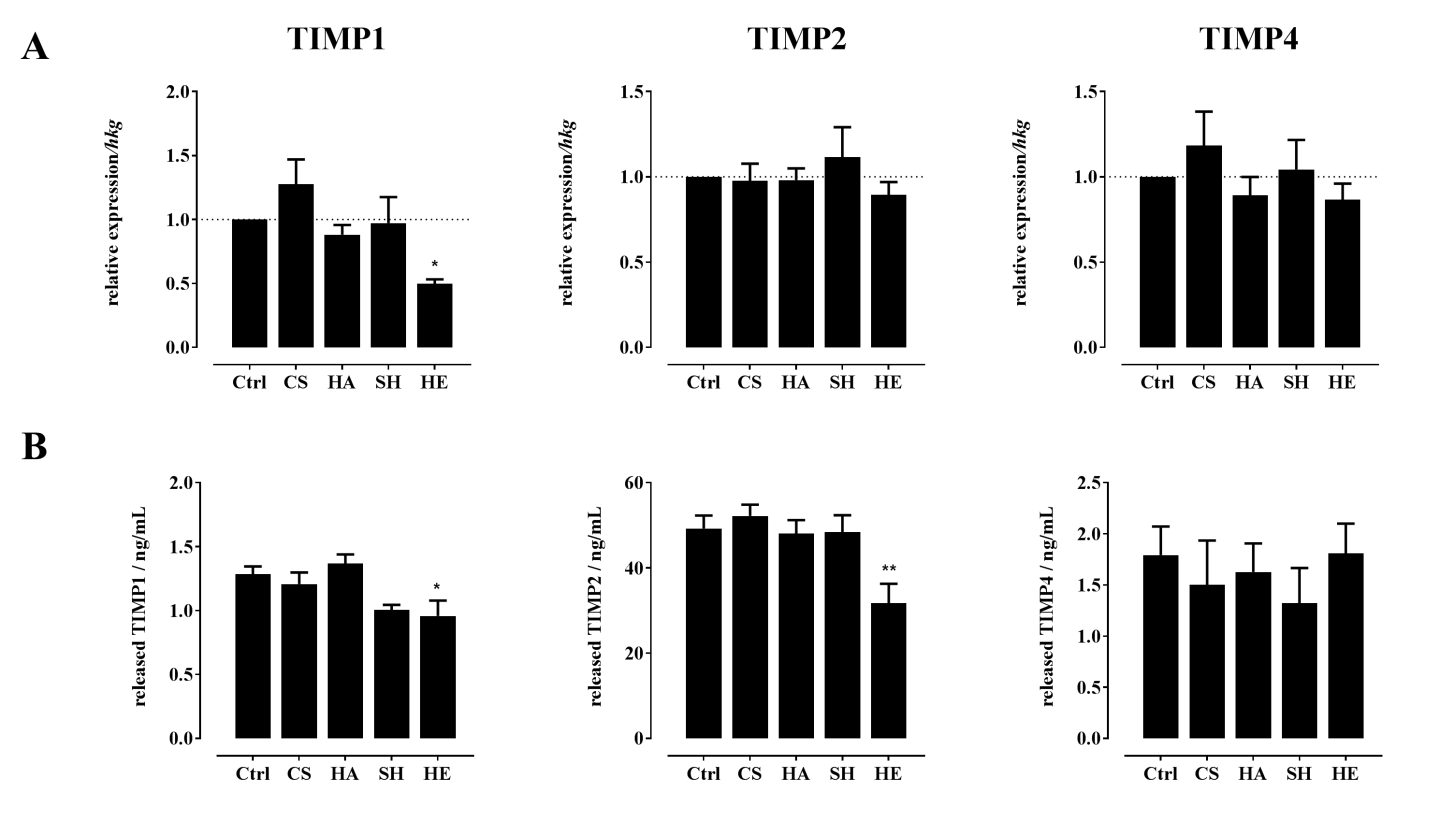
**

**Fig. S2: *Influence of GAG on RNA and protein level of TIMP1, TIMP2 and TIMP4 in hBMSC.***

7,000 hBMSC/cm² were plated in basic medium and treated with CS, HA, SH, and HE (200 µg/mL each). At day 22 after plating cells and conditioned medium were analyzed. (**A**) Gene expression of *timp1*, *timp2* and *timp4* was assessed by qPCR, normalized to the expression of the house-keeping genes (*hkg*) *gapdh*, *β-actin*, and *rps26*, and related to untreated control (Ctrl, set to 1) using the comparative quantitation method. Conditioned medium of hBMSC was analyzed for TIMP1, TIMP2 and TIMP4 protein contents using commercially ELISA Kits (**B**). The results are presented as mean ± SEM. Significant differences of treatment vs. Ctrl were analyzed by one-way ANOVA/Bonferroni’s post-test and are indicated with * (p<0.05) and ** (p<0.01), n=4.

**
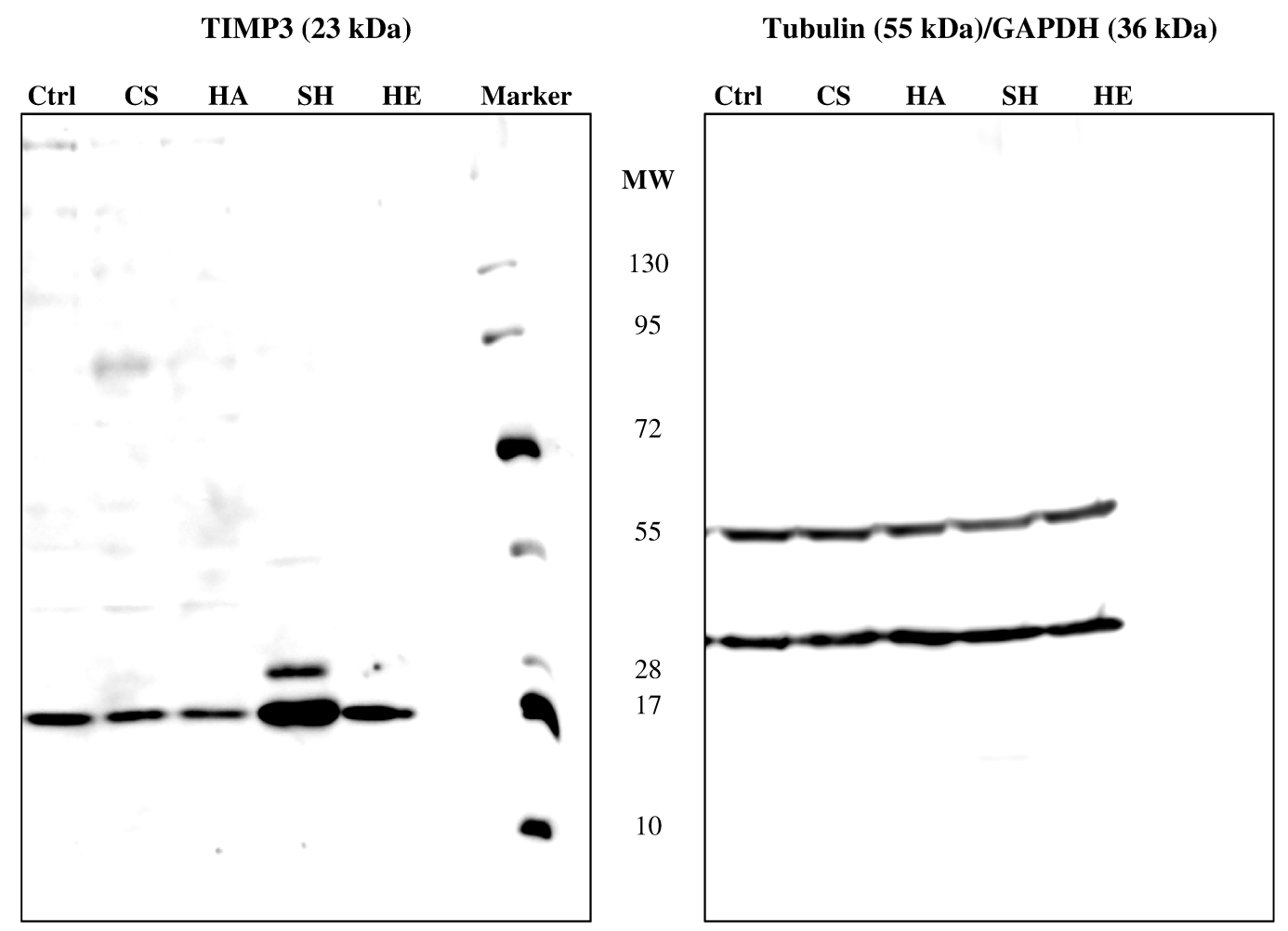
**

**Fig. S3: *Representative full length blot of TIMP3, reblotted with tubulin/GAPDH.***

The protein content of TIMP3 in hBMSC lysates was analyzed by Western blotting and related to the protein content of tubulin and GAPDH. (**A**) Image shows a representative full length membrane of the TIMP3 blot; the same membrane was afterwards reblotted with anti-tubulin/anti-GAPDH antibody mixture (**B**). For Fig. 1G the regions of interest were cropped and equalized for size.

| **A** | **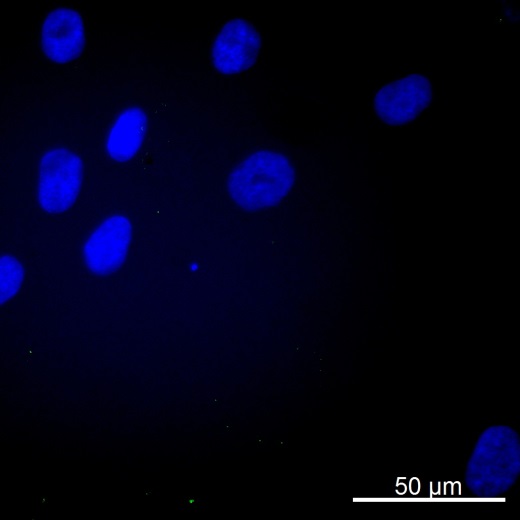** | **B** | **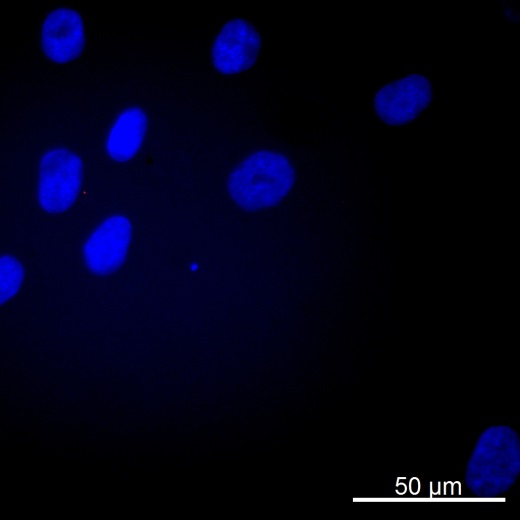** |
| --- | --- | --- | --- |

**Fig. S4: *Control stainings with secondary antibodies.***

hBMSC were cultivated for 8 days, fixed with paraformaldehyde and stained with secondary antibodies AlexaFluor488 rabbit anti-goat-IgG (**A**, green) and AlexaFluor568 goat anti-mouse-IgG (**B**, red). The staining was performed as described however without specific primary antibodies. Nuclei (blue) were stained with DAPI. Scale bars 50 µm.

**
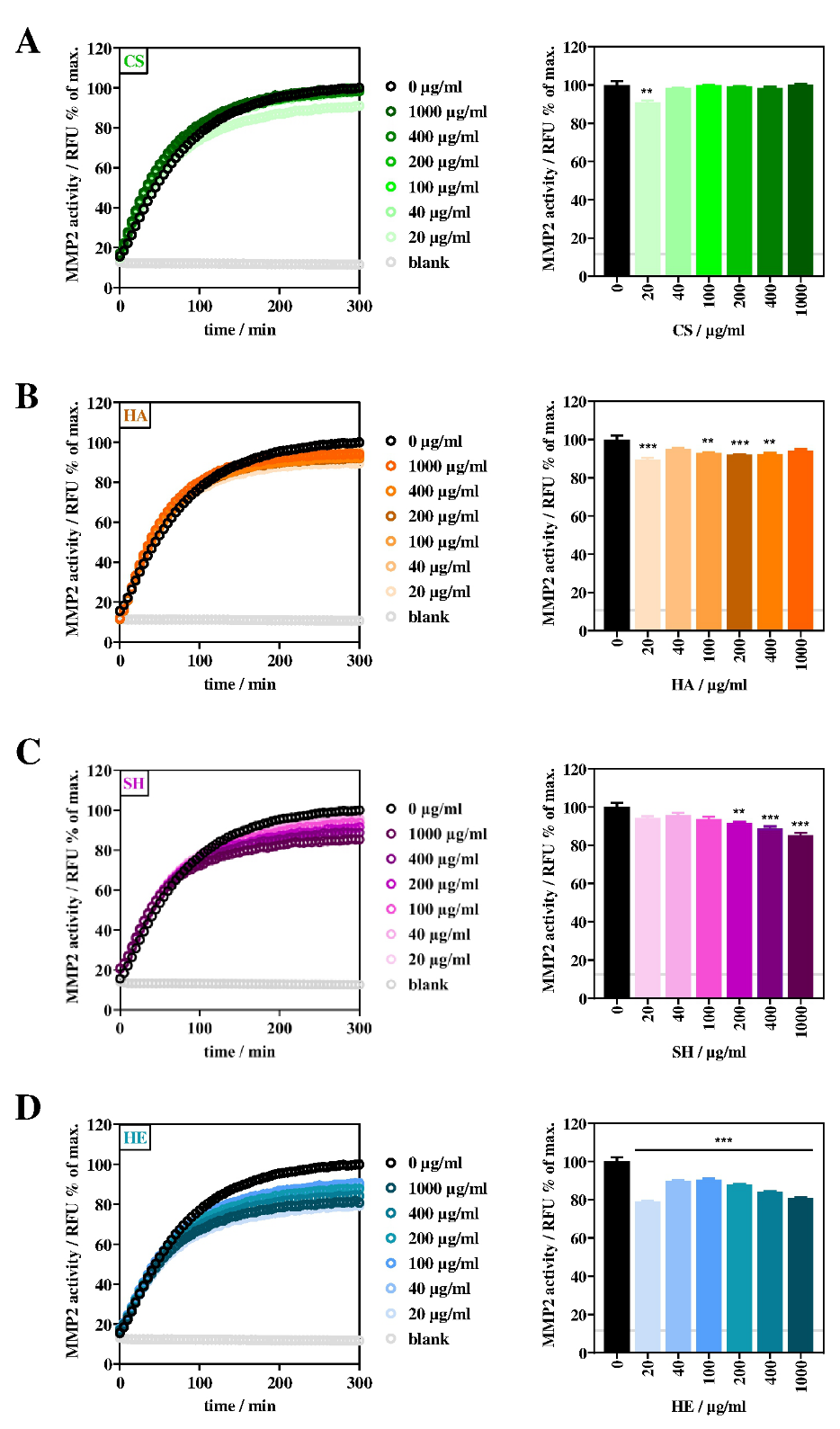
**

**Fig S5: Influence of CS, HA, SH, and HE on MMP2 enzyme activity.**

MMP2 enzyme activity was determined with rhMMP2 (100 ng/mL) and 50 µM fluorogenic peptide (MCA-Pro-Leu-Ala-Nva-Dpa-Ala-Arg-NH₂) as a substrate in the presence of 20-1,000 µg CS (**A**), HA (**B**), SH (**C**), and HE (**D**)/mL respectively. The diagrams show the MMP2 activity values obtained in the kinetics mode as percent of maximum activity after 5 h (left panel) and the endpoint values (right panel, the horizontal line indicates the blank value). The results (B) are presented as mean ± SEM. Significant differences of treatment vs. Ctrl (0 µg GAG/mL) were analyzed by One-way ANOVA/Bonferroni’s post-test and are indicated with ** (p<0.01) and *** (p<0.001).

**
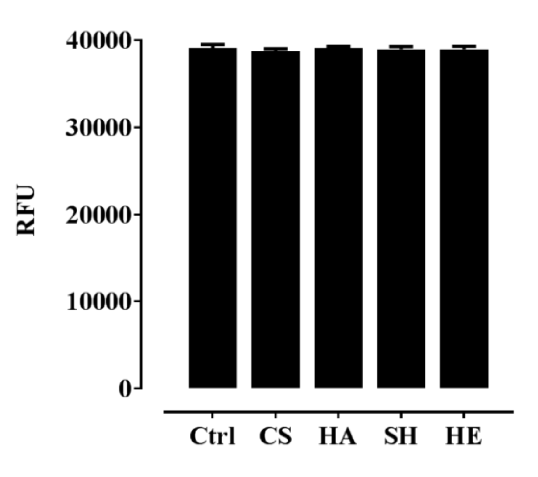
**

**Fig. S6: *Quenching effects of GAG on fluorescence signal.***

To see whether GAG itself caused a quenching of fluorescence signal, (200 µg of each GAG/ml were incubated with 50 µM of Mca-Pro-Leu-OH (fluorogenic control peptide, product of MMP2-induced MCA-Pro-Leu-Ala-Nva-Dpa-Ala-Arg-NH₂-cleavage) for 5 h and compared to Mca-Pro-Leu-OH without GAG. The results show the fluorescence signal after 5 h and are presented as mean ± SEM, n=4.


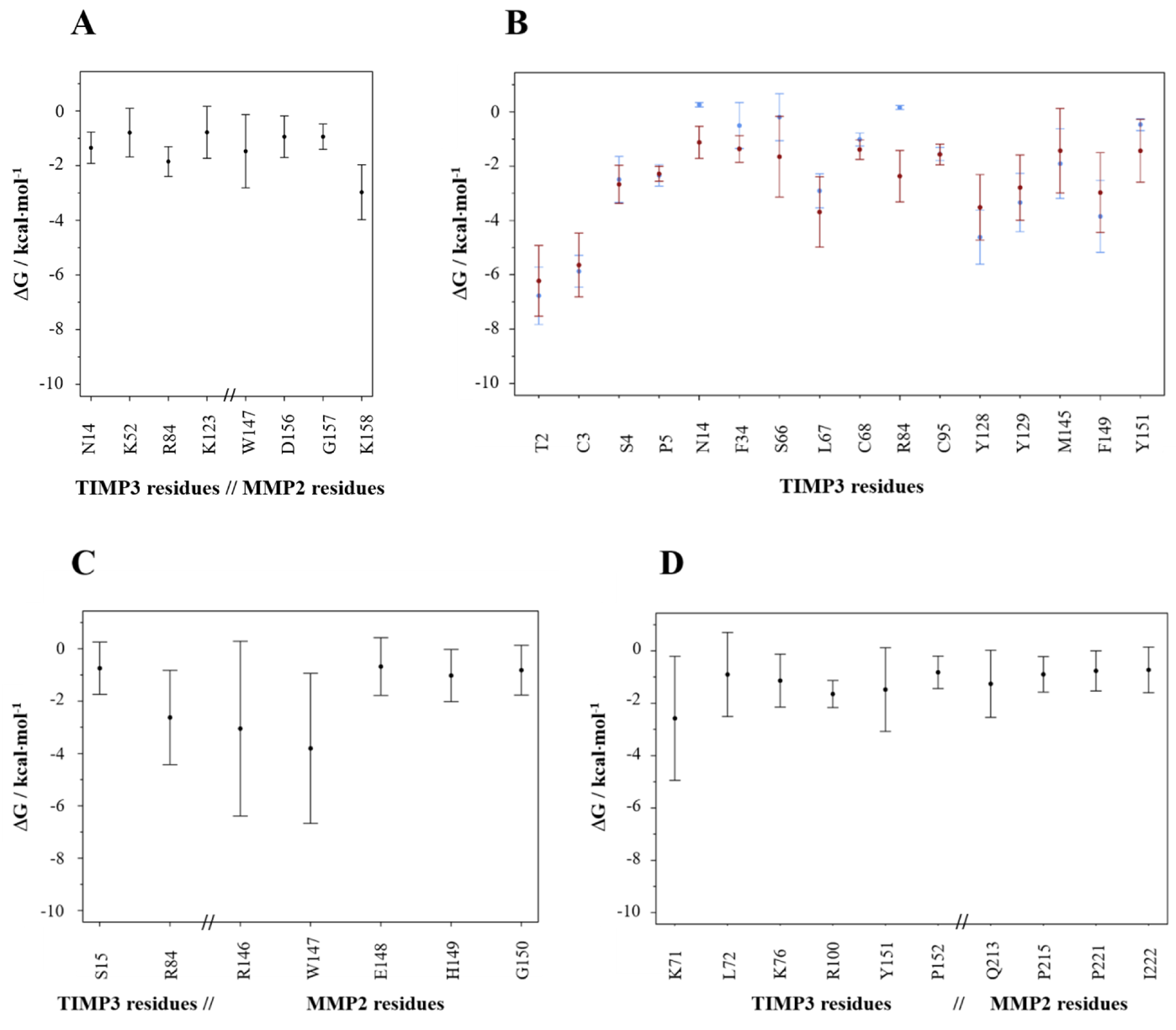


**Fig. S7: *Analysis of HA in complex with MMP2/TIMP3.***

Per-residue binding energy contribution calculated with MM-GBSA from four independent MD simulations of HA interacting with MMP2/TIMP3 complex (according to table 1 and Fig. 4F). (**A**) HA Cluster1, (**B**) TIMP3 in complex with MMP2 in the absence (blue) and presence (brown) of HA Cluster1, (**C**) HA Cluster2 and (**D**) HA Cluster3. The binding energy results are presented as mean ± SEM.

**
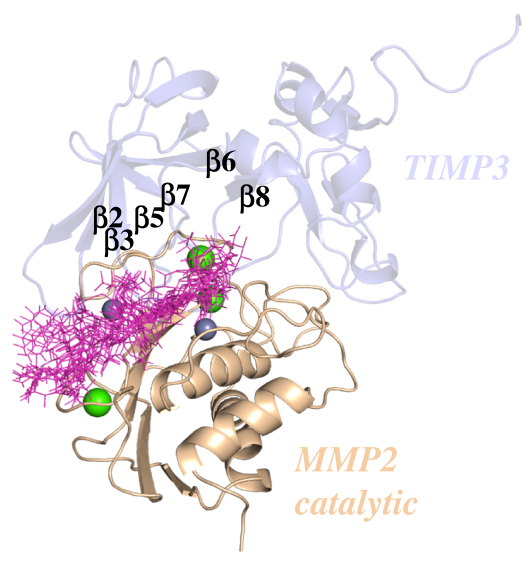
**

**Fig. S8: *Molecular modelling of SH in complex with MMP2.***

Docking results using Autodock3 and DBSCAN clustering are shown. MMP2 is shown in pale cartoon, and the SH cluster is depicted in pink sticks. Calcium and zinc ions are shown in green and grey spheres, respectively. TIMP-3 (not taken into account for docking) is shown in blue cartoon transparency. Turned view according to Fig. 4H.


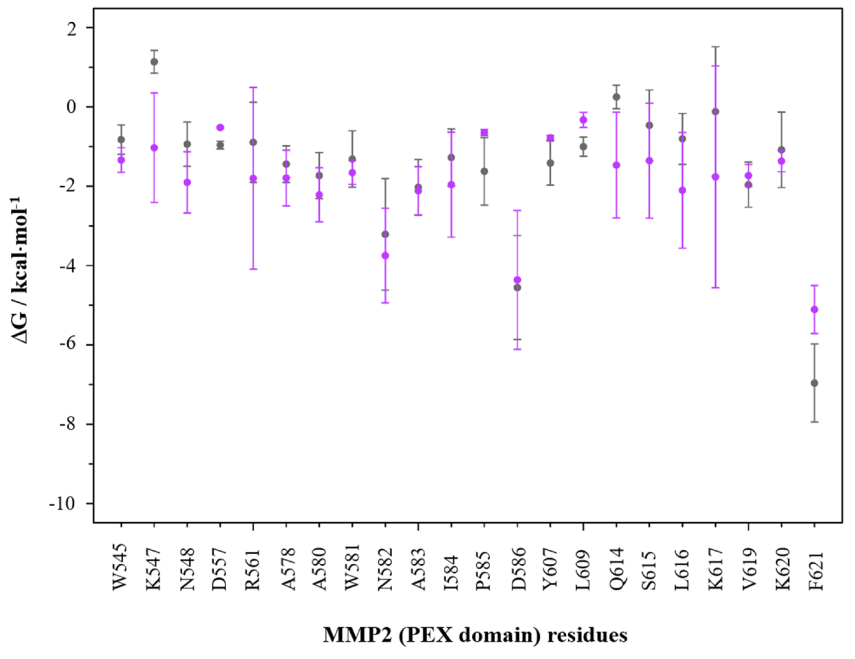


**Fig. S9: *Analysis of SH in complex with proMMP2(PEX domain)/TIMP3.***

Per-residue binding energy contribution of proMMP2(PEX domain)/TIMP3 complex calculated with MM-GBSA from four independent MD simulations in the absence (grey) and in the presence (pink) of SH (according to Fig. 6). The binding energy results are presented as mean ± SEM.
